# Social Interaction Affects Neural Outcomes of Sign Language Learning As a Foreign Language in Adults

**DOI:** 10.3389/fnhum.2017.00115

**Published:** 2017-03-31

**Authors:** Noriaki Yusa, Jungho Kim, Masatoshi Koizumi, Motoaki Sugiura, Ryuta Kawashima

**Affiliations:** ^1^Department of English, Miyagi Gakuin Women's UniversitySendai, Japan; ^2^Department of Foreign Languages, Kyoto Women's UniversityKyoto, Japan; ^3^Graduate School of Arts and Letters, Tohoku UniversitySendai, Japan; ^4^Institute of Development, Aging and Cancer, Tohoku UniversitySendai, Japan

**Keywords:** social interaction, foreign language learning, fMRI, Japanese sign language, syntax, left inferior frontal gyrus

## Abstract

Children naturally acquire a language in social contexts where they interact with their caregivers. Indeed, research shows that social interaction facilitates lexical and phonological development at the early stages of child language acquisition. It is not clear, however, whether the relationship between social interaction and learning applies to adult second language acquisition of syntactic rules. Does learning second language syntactic rules through social interactions with a native speaker or without such interactions impact behavior and the brain? The current study aims to answer this question. Adult Japanese participants learned a new foreign language, Japanese sign language (JSL), either through a native deaf signer or via DVDs. Neural correlates of acquiring new linguistic knowledge were investigated using functional magnetic resonance imaging (fMRI). The participants in each group were indistinguishable in terms of their behavioral data after the instruction. The fMRI data, however, revealed significant differences in the neural activities between two groups. Significant activations in the left inferior frontal gyrus (IFG) were found for the participants who learned JSL through interactions with the native signer. In contrast, no cortical activation change in the left IFG was found for the group who experienced the same visual input for the same duration via the DVD presentation. Given that the left IFG is involved in the syntactic processing of language, spoken or signed, learning through social interactions resulted in an fMRI signature typical of native speakers: activation of the left IFG. Thus, broadly speaking, availability of communicative interaction is necessary for second language acquisition and this results in observed changes in the brain.

## Introduction

It is a trivial fact that all normal children effortlessly acquire a particular language used around them. Less trivial is the fact that children do so through social interactions: children cannot acquire a language from linguistic input such as TV, or computer presentations (Sachs et al., 1981; Baker, 2001; Kuhl et al., 2003). This fact is all the more worth remarking, considering that other cognitive systems such as the visual system do not require human interaction for them to develop properly from birth. In this sense, language is uniquely human in that it is inherently social (de Saussure, 1916/1972).

In addition to the atypical cases of children raised in social isolation such as the wild boy of Aveyron (Lane, 1976) and Genie (Curtiss, 1977), the importance of a communicative partner in language acquisition has been illustrated by Sachs et al. (1981): the case of hearing children raised by deaf parents, who attempted in vain to teach them spoken English via television. Kuhl et al. (2003) provide more direct evidence for the experimental effects of social interactions on phonetic learning (discrimination) in a foreign language. Infants less than 6 months old of age can discriminate various speech contrasts in the world that do not exist in their mother tongues (Eimas et al., 1971; Werker and Tees, 1984), but they lose the discriminating ability between 6 and 12 months of age (Werker and Tees, 1984). During this period, they grow into “native listeners” from “universal listeners.” In Kuhl et al.'s experiment, 9-to-10-old month American babies were exposed to a new language, Mandarin Chinese, over 4–6 weeks through four different speakers of Mandarin Chinese or via televised recordings of Mandarin Chinese speakers. After exposure, the researchers performed a head-turn phonetic discrimination task of a Mandarin fricative-affricate contrast that does not exist in English. Only infants exposed to Mandarin Chinese speakers retained their sensitivity to distinguish the non-native Mandarin speech contrast and showed the same level of phonetic discrimination as native speakers of Mandarin Chinese. The result clearly indicates that phonetic learning is not triggered by simple exposure to linguistic input, but that infants must be exposed to a language in socially interactive situations to develop speech perception (Kuhl, 2007). TV programs or DVDs cannot be substitutes for human instruction in the early periods of phonetic learning.

Social interactions provide a variety of information needed for language development, so that several explanations have been offered for the findings in Kuhl et al. (2003). Social interactions may “attract more attention and increase motivation” in infants (Verga and Kotz, 2013, p. 3) resulting in phonetic learning; joint attention may provide more referential information needed for the association of a word and its referent (Kuhl et al., 2003); social contingency or back-and-forth feedback from humans may play a vital role in language development (Kuhl, 2007; Roseberry et al., 2014); infants may not be familiar or experienced with DVD presentations. These explanations are not mutually exclusive or implausible in that infants acquire a language through social interactions with their caregivers that involve child-directed speech (Bruner, 1983). The reader is referred to Hoff (2006) and Verga and Kotz (2013) for the review of relevant studies showing that social interaction influences language learning in infants.

Despite the alleged importance of social interaction in language development, previous language learning studies on social interaction only focused on vocabulary learning (Kuhl, 2007) and phonetic discrimination (Kuhl et al., 2003; Kuhl, 2007) in a foreign language during childhood, and word learning in a first language (Krcmar et al., 2007; Roseberry et al., 2009; Verga and Kotz, 2013). Language is, however, more than words and sounds. Human language is a computational system of connecting meaning and sound (or a visual-manual channel in sign languages) by means of syntactic structure. Syntactic structure has not been observed in other species (Hauser et al., 2002), so structure dependence in this sense is the most characterizing feature of human language (Chomsky, 2013a,b; Everaert et al., 2015; Berwick and Chomsky, 2016).

In spite of the fact that syntax is “the basic property” of human language (Chomsky, 2013a,b) and that social interaction plays a key role in early language development, syntax has not been discussed in adult second language acquisition research from the perspective of social interaction. It is true that the number of neuroscience studies on social interactions has exponentially increased over the last decade (for a research review, see Verga and Kotz, 2013), little social neuroscience research has until recently dealt with adult second language acquisition. One of the few studies on second language acquisition in different social settings is Jeong et al. (2010). Jeong and her colleagues tested the effects of social interactions on the acquisition of second language vocabulary by adult learners. They compared the retrieval of words learned from text-based learning (written translations) and that of words learned from situation-based learning (real-life situations). The result shows that the comprehension of words learned through movie-clips depicting a social situation elicited activity in the right supramarginal gyrus similar to that evoked by the comprehension of vocabulary in one's native language. The result indicates the effects of social interaction in second language acquisition of vocabulary on the brain, but it should be noted that participants in situation-based learning contexts learned foreign language vocabulary through “artificial” movie-clips of a dialogue. Therefore, it remains to be elucidated what differences *natural* social interaction with a teacher makes in second language acquisition in comparison to learning a second language with artificial interaction such as DVDs (Verga and Kotz, 2013). In fact, no study, to our knowledge, has yet investigated how social interaction during foreign language learning in adulthood will affect neural mechanisms. Thus, whether adult learners benefit from learning in social contexts is still an open issue.

Given that linguistic knowledge is internalized in the brain and that more people are using computer-assisted learning without human interaction, we reasonably address the non-trivial question of whether social interaction will have distinctive effects on the brains of adults learning foreign language syntax, which is more complex than vocabulary and phonetic learning. Due to resource constraints, non-interactive learning through a combination of audio and video is common among second language learners who have few opportunities to interact with native speakers of a target language. It should be noted, however, that there is no clear evidence that computer-supported learning without social interaction has the same effects on the learning of syntactic rules in a foreign language as learning through human interaction. Most studies on social interactions are based on behavior or performance data, but behavioral data have some limitations. First, behavioral scores of linguistic knowledge are blurred by numerous factors such as attention, cognition, and perception. It is, therefore, extremely difficult, if not impossible, to tease them apart, which in turn makes the interpretation of the performance data inconclusive (Raizada et al., 2008). Second, behavioral data do not reveal the neurocognitive mechanisms responsible for the processing of second language knowledge. Third, similar behavioral data do “not necessarily implicate reliance on similar neural mechanisms” (Morgan-Short et al., 2012, p. 934). Indeed, several brain imaging studies (Musso et al., 2003; Osterhout et al., 2008; Sakai et al., 2009) have reported the evidence for the difference between performance data and their respective neuroscience data.

The present paper discusses whether presence or absence of a human being has distinct effects on neural (fMRI) and behavioral (performance) measures of syntactic processing of a foreign language in adults. As a foreign language, we tested the acquisition of Japanese sign language (JSL) by Japanese adults who had not learned JSL. A sign language is mistakenly conceived to be a kind of artificial pantomime-like gesture lacking linguistic structure or at least a variant of a spoken language, but neither is well-grounded. A great deal of research in recent years demonstrates that sign language is a natural language with rich grammatical properties that characterize other natural languages such as spoken English or Japanese (Sandler and Lillo-Martin, 2006). Cecchetto et al. (2012), for example, demonstrate that Italian sign language respects structure dependence based on abstract hierarchical syntactic structure characteristic of only human languages (Chomsky, 2013a,b; Everaert et al., 2015). Studies of language development have also provided evidence that deaf children experience almost the same stages of language development as hearing children (Petitto and Marentette, 1991). Deaf babies, for instance, experience a stage of manual babbling during the same period as hearing children go through a stage of vocalization babbling. This confirms that irrespective of superficial speech modality differences, the same mechanism applies to core functions of sign and spoken languages. Differences between the two languages derive from the modalities in which they are produced and comprehended (MacSweeney et al., 2008). Furthermore, neuroimaging studies show that comprehension of spoken and sign languages activates the classical language brain regions including the left inferior frontal gyrus (IFG) (Sakai et al., 2005) in addition to the left superior temporal gyrus and sulcus (for a relevant literature review, see MacSweeney et al., 2008).

Areas in the left IFG, specifically the posterior pars opercularis (BA 44) and the more anterior pars triangularis (BA 45) of Broca's area, are known to be involved in processing linguistic and non-linguistic information (e.g., Koechlin and Jubault, 2006; Tettamanti and Weniger, 2006). This leads to the suggestion that Broca's area works as a “supramodal processor of hierarchical structures” (Tettamanti and Weniger, 2006). The “supramodal syntactic processor” (Clerget et al., 2013) has been localized either in BA 44 (Bahlmann et al., 2009; Fazio et al., 2009) or in BA 45 (Santi and Grodzinsky, 2010; Pallier et al., 2011). We will not go into the issue of which region, BA 44 or BA 45, is selectively responsible for processing syntactic structure (Musso et al., 2003; Pallier et al., 2011; Yusa, 2012; Goucha and Friederici, 2015; Zaccarella and Friederici, 2015; Zaccarella et al., 2017), but instead follow previous research showing that syntactic processing in a first language and a second language activates Broca's area in the left IFG (Perani and Abutalebi, 2005; Abutalebi, 2008). In particular, syntactic rules satisfying structure dependence selectively activate the language area of the brain, specifically the left IFG, while syntactic rules violating structure-dependent rules do not (Musso et al., 2003; Yusa et al., 2011). In addition, instruction effects of syntax in a second language are reflected in the left IFG (Musso et al., 2003; Sakai et al., 2009; Yusa et al., 2011). Recent extensive research on syntax processing also validates the claim that the left IFG is responsible for processing syntactic structure (Moro et al., 2001; Musso et al., 2003; Friederici et al., 2011; Goucha and Friederici, 2015). All taken together, we assume that activation of the left IFG is indicative of the acquisition of syntactic rules respecting structure-dependence.

We show, by examining the acquisition of JSL under two different social learning conditions, that learning through interaction with a deaf signer resulted in a stronger activation of the left IFG than learning through identical input via DVD presentations, though behavioral data did not show distinct differences.

## Japanese sign language

JSL has the basic word or constituent order of SOV (subject-object-verb), but exhibits free word order as spoken Japanese does. The basic word order SOV can be changed into its topicalized order OSV with the topicalized O accompanied by a set of non-manual markers (NMM) such as eyebrow raising and nodding. There are, however, some restrictions on constituent order. Consider the following wh-cleft sentence “/PT-I/ /FATHER/ /OCCUPATION/ /WHAT/ /DOCTOR/,” which means “What my father is is a doctor.” (Following conventions, signs are written as glosses in capital letters and PT stands for “pointing to the nose or chest with the index finger of either hand”). In JSL, possessives cannot be moved from their modifying head nouns, whose phenomenon in spoken languages has been discussed in terms of the Left Branch Condition since Ross (1967). We call this the Possessive Construction Restriction. For example, possessive pronoun MY indicated by /PT-I/ cannot be separated from *FATHER* as in “/FATHER/ /OCCUPATION/ /PT-I/ /WHAT/ /DOCTOR/,” which is ungrammatical. Although languages differ as to whether they allow left-branch extraction (Bošković, 2005), it suffices to note for the purpose of the present paper that left-branch extraction is disallowed in JSL. What matters here is the syntactic difference between the optionality of topicalization of objects and the prohibition of the movement of possessives from their modifying nouns. In this sense, movement of a constituent respects structure dependence in a sense that movement of a constituent depends on the syntactic structure of the moved constituent.

It is interesting to note at this point that even a native speaker of JSL in our experiment had not considered the possessive construction restriction until it was pointed out, so it is natural that no book on JSL we know of refers to any aspects of the possessive construction restriction.

## Materials and methods

### Participants

Forty six adult Japanese without any knowledge of JSL participated in our experiment. Participants were all recruited from Miyagi Gakuin Women's University, Sendai, Japan. They were divided into the Live-Exposure Group and the DVD-Exposure Group on the basis of working memory measured by the reading span test. As a result, those groups were indistinguishable on working memory before JSL lessons (*t*_(44)_ = 0.249, *p* = 0.80). Before the experiment, all participants were provided with minute explanations of the experiment and its safety. They gave written informed consent for the study and right-handedness was verified using the Edinburgh Inventory (Oldfield, 1971). All experiments were performed in compliance with the relevant institutional guidelines approved by Tohoku University. Approval for the study was obtained from the Ethics Committee of the Institute of Development, Aging and Cancer, Tohoku University.

### Procedure and stimuli

The Live-Exposure Group and the DVD-Exposure Group learned JSL in two different contexts. Twenty two participants in the Live-Exposure Group learned JSL through social interactions with a native signer of JSL in ten 80-min classes in 1 month, where they learned the JSL expressions related to self-introduction, numbers, family, transportation, weather, hobbies, food and so on. A native signer did not teach the participants the grammar of JSL, but a large number of expressions in JSL in an implicit way. On the other hand, 24 participants in the DVD-Exposure Group learned JSL in the same number of classes during the same period through the DVDs that recorded the class lessons in the Live-Exposure Group. Therefore, the difference between the Live-Exposure Group and the DVD-Exposure Group was the existence/absence of social interchanges through a deaf signer.

**Figure 1:**
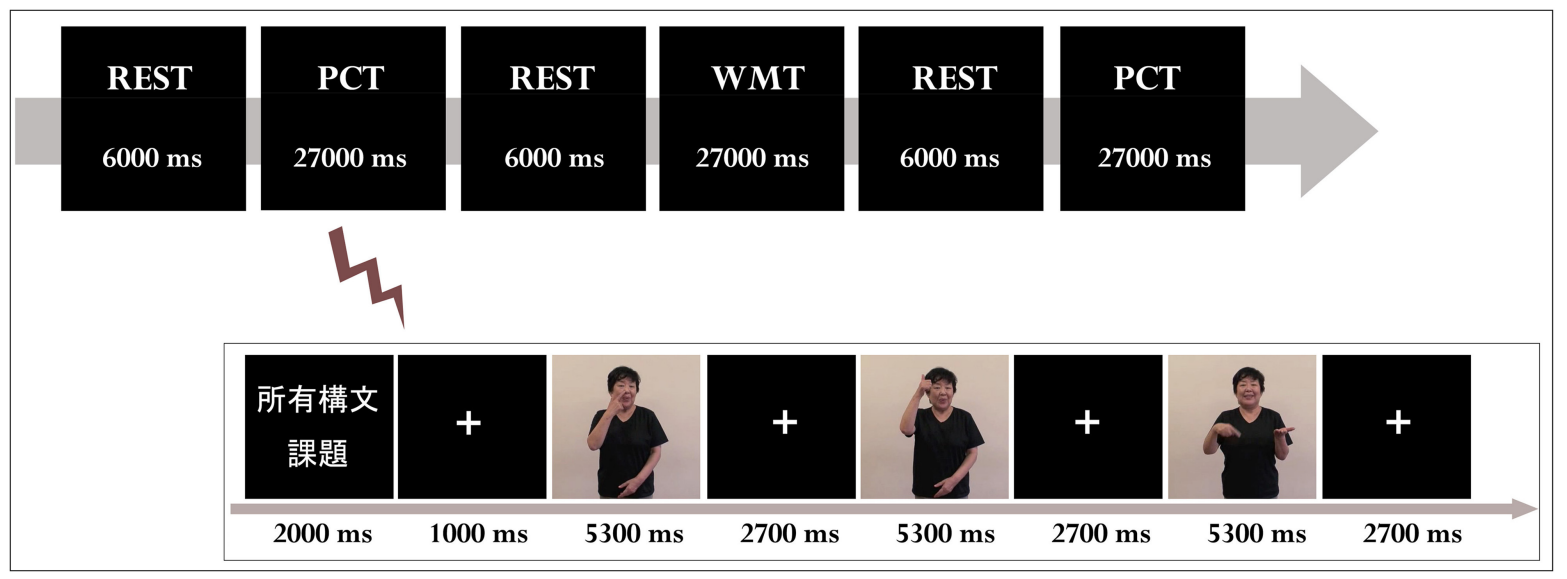
**Timeline in the experimental task**. PCT, Possessive Construction Task; WMT, Working Memory Task; REST, Rest Task. The experiment was performed in a block design. Participants were asked to judge whether the JSL they saw on the screen was correct. Response time was recorded from the beginning of each stimulus sentence until the button was pressed. E-prime ver. 2.0 (Psychology Software Tools) was used to present the stimuli and obtain the behavioral data.

The participants in both groups underwent two sets of fMRI measurements after the 4th class (TEST 1) and the 10th class (TEST 2). Stimuli were visually presented to the participants in a block design (Figure 1). The total number of stimuli was 72, which was divided into two sessions with 36 stimuli each. Each session consisted of three conditions: Possessive Construction Task (correct/incorrect), Working Memory Task (correct/incorrect), and Rest Task (Table 1). In the Possessive Construction Task (PCT), the participants were visually presented with both possible and impossible JSL in random order on a screen; they had to judge the grammaticality of the JSL by pressing a button. The second task was the Working Memory Task (WMT): the participants were presented with three signs in sequence and had to judge whether the sequence included three different signs: A stimulus with three different signs was judged as a “grammatical JSL,” whereas a stimulus involving two identical signs was regarded as an “ungrammatical JSL.”

**Table 1:** **Sample stimuli used in the fMRI experiment**.

Possessive Construction Task (PCT)
What my father is is a doctor.
Grammatical JSL /PT-I/ /FATHER/ /OCCUPATION/ /WHAT/ /DOCTOR/ “What my father is is a doctor” Ungrammatical JSL /FATHER/ /OCCUPATION/ /PT-I/ /WHAT/ /DOCTOR/ “What my father is is a doctor”
Working Memory Task (WMT)
Grammatical JSL /JOB/ /EAT/ /STUDY/ Ungrammatical JSL /WRITE/ /READ/ /WRITE/

*PT; Pointing Sign*.

*Japanese Sign language (JSL) has the basic word or constituent order of SOV (subject-object-verb), but exhibits free word order as spoken Japanese does. There are, however, some restrictions on constituent order. Consider “/PT-I/ /FATHER/ /OCCUPATION/ /WHAT/ /DOCTOR/,” which means “What my father is is a doctor.” In JSL, possessives cannot be moved from their modifying head nouns, whose phenomenon in spoken languages has been discussed in terms of the Left Branch Condition since Ross (1967). For example, possessive pronoun MY indicated by /PT-I/ cannot be separated from FATHER as in “/FATHER/ /OCCUPATION/ /PT-I/ /WHAT/ /DOCTOR/,” which is ungrammatical. In the Possessive Construction Task (PCT), where the participants were visually presented with both possible and impossible JSL in random order on a screen, they had to judge the grammaticality of the JSL by pressing a button. In the Working Memory Task (WMT), the participants were instructed to determine whether the sequence just presented included three different signs. A stimulus with different three signs was treated as a “grammatical JSL” stimulus, whereas a stimulus including two identical signs was regarded as an “ungrammatical JSL” sequence*.

The Rest Task (REST) required the participants to gaze at a fixation cross. All stimuli were controlled using E-prime ver. 2.0 (Psychology Software Tools). Figure 1 shows how the experiments proceeded. Following Hashimoto and Sakai (2002), we employed the WMT in our experiment. Its rationale was to disassociate working memory effects from the comprehension of JSL. The comprehension of a language is based on structure-dependent operations. Moreover, language comprehension is incremental in that linguistic information of a lexical item is processed immediately every time it is encountered (Neville et al., 1991; Phillips, 2003). Therefore, the PCM task implicitly required the participants to encode linguistic information of signs and decode it from working memory when they judged the JSL.

### Image acquisition

Functional neuroimaging data were acquired with a 3.0 Tesla MRI scanner (Philips Achieva Quasar Dual, Philips Medical Systems, Best, The Netherlands) using a gradient echo planar image (EPI) sequence ([TE] = 30 ms, field of view [FOV] = 192 mm, flip angle [FA] = 70°, slice thickness = 5 mm, slice gap = 0 mm). Thirty-two axial slices spanning the entire brain were obtained every 2 s. After the attainment of functional imaging, T1-weighted anatomical images were also acquired from each participant.

### Analysis

All data processing and group analyses were performed using MATLAB (The Mathworks Inc., Natick, MA, USA) and SPM8 (Wellcome Department of Cognitive Neurology, London, UK). The acquisition timing of each slice was corrected using the middle (16th in time) slice as a reference for EPI data. In order to correct for head movement artifacts, functional images were first resliced and subsequently realigned with the first scan of the subjects. After alignment to the AC-PC line, each participant's T1-weighted image was coregistered to the mean functional EPI image and segmented using the standard tissue probability maps provided in SPM8. The coregistered structural image was spatially normalized to the Montreal Neurological Institute (MNI) standard brain template. All normalized functional images were then smoothed with a Gaussian kernel of 8 mm full-width at half-maximum (FWHM). An analysis of the tasks for each participant was conducted at the first statistical stage and a group statistical analysis at the second stage. Contrasts in the PCT – WMT condition was calculated using a one sample *t*-test. The threshold for significant activation of each contrast was set at *p* < 0.001, uncorrected. The spatial extent threshold was set at *k* = 10 voxels. Finally, we performed a region of interest (ROI) analysis in the brain area obtained from the comparison [PCT – WMT_(TEST 2)_] – [PCT – WMT _(TEST 1)_]. Activation maxima are reported as MNI-coordinates and anatomical regions are based on the Talairach Client (Lancaster and Fox, Research Imaging Center, University of Texas Health Science Center San Antonio; Talairach and Tournoux, 1988; Lancaster et al., 2000).

### Predictions

If linguistic input is sufficient to induce JSL learning in adults, then exposure to JSL via a deaf signer or DVDs should result in the same changes in behavioral and imaging data. Instead, if social interaction is required and is an important factor in JSL learning, the Live-Exposure Group and the DVD-Exposure Group should show a different pattern of activation in the brain, or more specifically, the former group should have greater activation in the left IFG than the latter group.

## Results

All data processing analyses were performed using SPM8. The threshold for significant activation of each contrast was set at *p* < 0.001, uncorrected. We analyzed data from 18 participants (mean age ± SD: 20.7 ± 0.76 years) in the Live-Exposure Group and 21 participants (mean age ± SD: 20.6 ± 0.76 years) in the DVD-Exposure Group. There was no significant difference in the percentage of error rates in TEST 1 or TEST 2 between the Live-Exposure Group and the DVD-Exposure Group [TEST 1, *t*_(37)_ = −0.62, *p* = 0.54; TEST 2, *t*_(37)_ = −1.71, *p* = 0.09] (Table 2). The result indicates that the participants in both groups developed the same level of knowledge of the Possessive Construction at the 4th and 10th trainings; their performance or behavior results were not significantly different. No significant difference in reaction times was observed in TEST 1 or TEST 2 between the Live-Exposure Group and the DVD-Exposure Group, either [TEST 1, *t*_(37)_ = −1.26, *p* = 0.22; TEST 2, *t*_(37)_ = −1.09, *p* = 0.28].

**Table 2:** **Error rates (%) and reaction times (ms) for PCT**.

	**Error rates (%)**		**Reaction times (ms)**	
	**TEST 1**	**TEST 2**	**TEST 1**	**TEST 2**
DVD-Exposure Group	42.5 (11.7)	26.2 (19.5)	6,117 (494)	5,984 (583)
Live-Exposure Group	39.1 (21.2)	15.5 (19.2)	5,899 (588)	5,788 (524)
	*p* = 0.54, *ns*	*p* = 0.09, *ns*	*p* = 0.22, *ns*	*p* = 0.28, *ns*

*There was no significant difference in the percentage of error rates and reaction times in TEST 1 or TEST 2 between the Live-Exposure Group and the DVD-Exposure Group. A significant performance improvement was, however, found in the DVD-Exposure Group as well as the Live-Exposure Group between TEST 1 and TEST 2: the percentage of errors in TEST 2 significantly decreased with both groups as compared to that in TEST 1 [Live-Exposure Group; t_(17)_ = 4.79, p < 0.001; DVD-Exposure Group; t_(20)_ = 4.82, p < 0.001]*.

A significant performance improvement was, however, found in the DVD-Exposure Group as well as the Live-Exposure Group between TEST 1 and TEST 2 (Table 2): the percentage of errors in TEST2 significantly decreased with both groups as compared to that in TEST 1, indicating that teaching JSL through a native signer or DVDs had significant effects on the acquisition of the Possessive Construction Restriction [Live-Exposure Group; *t*_(17)_ = 4.79, *p* < 0.001; DVD-Exposure Group; *t*_(20)_ = 4.82, *p* < 0.001].

### Imaging data

To identify cortical activation generated in two different learning contexts (i.e., via social interactions with a deaf signer and through DVDs), we subtracted [PCT – WMT_(TEST 1)_] from [PCT – WMT_(TEST 2)_]. Table 3 shows the activated regions in the comparison of [PCT – WMT_(TEST 2)_] – [PCT – WMT _(TEST 1)_]. For the DVD-Exposure Group, we found increased activations in the right middle frontal gyrus, the bilateral cuneus, the right superior temporal gyrus, the right middle temporal gyrus, the right IFG. For the Live-Exposure Group, activations in the right parietal lobule, the right IFG, the right middle frontal gyrus, the left IFG, the left Inferior parietal gyrus, and the middle frontal gyrus increased.

**Table 3:** **Activated regions in the contrast [PCT – WMT_(TEST 2)_] – [PCT – WMT _(TEST 1)_]**.

**Hemisphere**	**Anatomical region**	**MNI**			***t*****-value**
		***x***	***y***	***z***	
**DVD-EXPOSURE GROUP**					
R	Medial frontal gyrus (BA 9)	9	44	25	5.92
R	Cuneus (BA 7)	9	−70	31	5.52
R	Cuneus (BA 7)	3	−64	31	4.49
L	Cuneus (BA 18)	−9	−76	28	4.38
R	Superior temporal gyrus (BA 39)	36	−49	28	4.99
R	Middle temporal gyrus (BA 39)	33	−61	31	4.51
R	Supramarginal gyrus (BA 40)	45	−49	34	4.32
R	Inferior frontal gyrus (BA 9)	45	11	31	4.85
R	Middle frontal gyrus (BA 9)	36	17	34	4.37
R	Midbrain	6	−13	−5	4.08
R	Thalamus	3	−4	4	3.82
**LIVE-EXPOSURE GROUP**					
R	Superior parietal lobule (BA 7)	30	−58	40	6.11
R	Precuneus (BA 7)	15	−64	37	3.89
R	Inferior frontal gyrus (BA 46)	45	38	10	5.66
R	Middle frontal gyrus (BA46)	42	47	16	4.47
R	Midbrain	9	−13	−11	5.17
R	Midbrain	3	−16	−17	4.72
L	Inferior frontal gyrus (BA 44)	−57	14	13	4.88
L	Inferior parietal lobule (BA 40)	−42	−52	40	4.65
R	Middle frontal gyrus (BA 9)	36	20	25	4.20
R	Middle frontal gyrus (BA 9)	45	8	37	4.15
R	Middle frontal gyrus (BA 9)	48	14	31	4.06

*Respective activated anatomic region, approximate Brodmann's area, right or left (R, L), t-values. Stereotactic coordinates (x, y, z) as defined by MNI are shown for each voxel with a local maximum of t-values in the contrasts indicated (p < 0.001, uncorrected)*.

**Figure 2:**
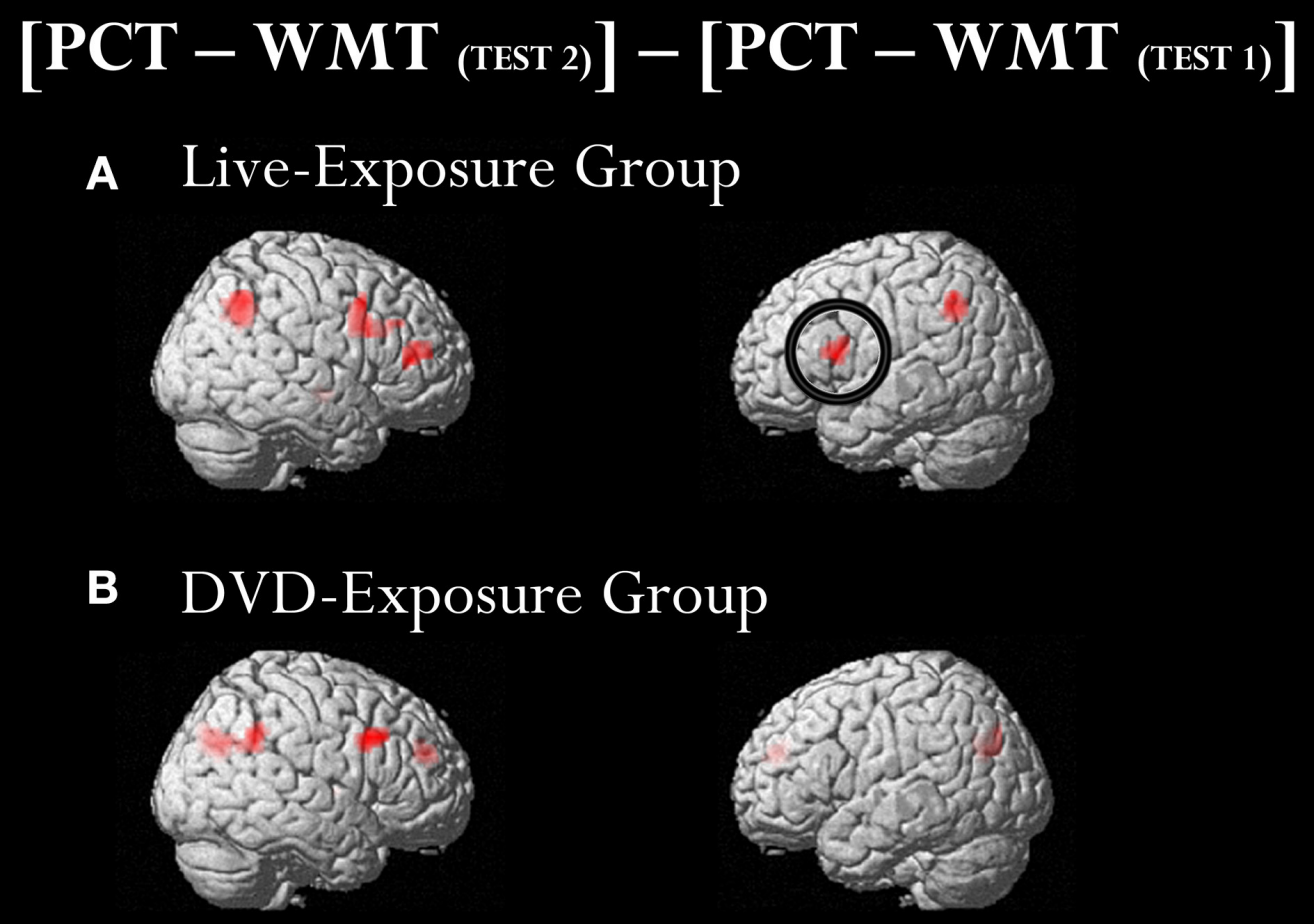
**Brain activated regions in the contrast [PCT – WMT_**(TEST 2)**_] − [PCT – WMT_**(TEST 1)**_]**. The participants in both groups underwent two sets of fMRI measurements after the 4th class (TEST 1) and the 10th class (TEST 2). To identify cortical activation generated after the instruction, we subtracted [PCT − WMT _(TEST 1)_] from [PCT − WMT_(TEST 2)_]. Significant activations in the left inferior frontal gyrus (IFG) were found only for the Live-Exposure Group **(A)**. No significant cortical activation change, by contrast, was found for the DVD-Exposure Group, who experienced the same visual input for the same duration via the DVD presentations **(B)**.

**Figure 3:**
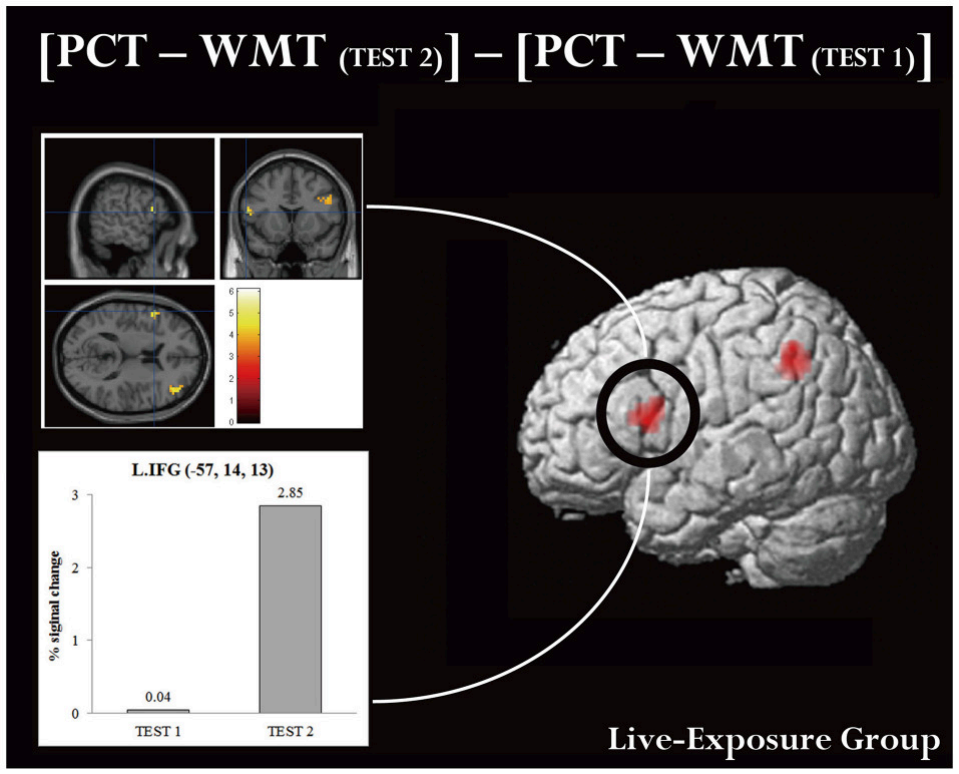
**Brain activation in MNI space and ROI analysis for the left IFG**. An ROI analysis was conducted in the left IFG, which is assumed to be involved in the processing of language. **(Upper panel)** cortical activation in [PCT – WMT_(TEST 2)_] – [PCT – WMT _(*TEST* 1)_] condition. **(Lower panel)** histograms for averaged maximum amplitudes of fitted hemodynamic responses at the local maximum in the left IFG. Each bar represents signal changes for TEST 1 and TEST 2, respectively. Note that signal changes in TEST 2 were significantly larger than in TEST 1 [*t*_(17)_ = −4.88, *p* < 0.001, *d* = −0.89].

A ROI analysis of each cluster was conducted using the SPSS 19 (SPSS Inc., IBM, Armonk, NY, USA) on the value of the single voxel of the peak coordinate, which was obtained using an in-house SPM-compatible MATLAB script. The ROI was set at the activated area in the contrast [PCT – WMT_(TEST 2)_] – [PCT – WMT_(TEST 1)_] pooling the data from two groups. Activity in this ROI was compared in each group between [PCT – WMT_(TEST 1)_] and [PCT – WMT_(TEST 2)_] using a paired *t*-test. Significant activations in the left IFG, an area assumed to be involved in the processing of syntactic rules (Musso et al., 2003; Abutalebi, 2008; Yusa, 2012; Zaccarella et al., 2017), were found only for the Live-Exposure Group [paired *t*-test: *t*_(17)_ = −4.88, *p* < 0.001]. No significant cortical activation change in the left IFG, by contrast, was found for the DVD-Exposure Group, who experienced the same visual input for the same duration via the DVD presentations [paired *t*-test: *t*_(20)_ = −0.29, *p* = 0.78, *n.s*.] (Figures 2, 3; Table 3). This result shows that (superficially) similar performance between the groups “does not necessarily implicate reliance on similar neural mechanisms” (Morgan-Short et al., 2012, p. 934). Given that the LIFG is involved in the syntactic processing of language, spoken or signed, only training in an interactional setting resulted in an fMRI signature typical of native speakers: activation of the left IFG.

## Discussion

The aim of the current investigation was to investigate the effects of social interaction on JSL learning in adult speakers. To examine social impacts on learning, we set up two types of learning contexts (that is, learning JSL through a deaf signer or through DVDs). Our results show that participants learned JSL equally in terms of behavioral data in both contexts, but that social interaction caused significant changes in the brain, particularly in the left IFG. This suggests that in addition to early speech learning in infants (Kuhl, 2007), social interaction is crucial in order for adult second language learners to come to rely on native-like neural mechanisms in processing syntactic rules or their efficient use. Social interaction through the interchanges with a deaf native signer may make it easier to “crack the JSL code,” neurologically supporting the view that language is inherently social (de Saussure, 1916/1972). Thus, learning accompanied by changes in brain functions is not triggered solely by linguistic input such as DVDs, but is enhanced by social interaction. The current research provides a significant platform for studies on second language learning in adults: linguistic input is necessary for second language learning, but influences of a social partner are different from the ones exerted from the source without social interactions.

Numerous studies reveal that JSL has linguistic characteristics distinct from spoken Japanese (Fischer, 1996, 2017; Matsuoka, 2015), which are to be discussed below. One might, however, object that the participants in our experiment simply transferred the knowledge of the possessive construction restriction in JSL from spoken Japanese, since extraction of possessives from their modifying nouns is also prohibited in spoken Japanese. This objection is plausible in light of the finding that in bilingualism both languages unconsciously influence each other (Kroll et al., 2006; Jarvis and Pavlenko, 2008), but it cannot explain why only the Live-Exposure Group experienced functional changes in the left IFG. If transfer from spoken Japanese had been a crucial factor in the learning of JSL in our experiment, learning JSL via DVD presentations would also have elicited similar activity in the left IFG. However, the lack of activation in the left IFG in the DVD Group rules out this possibility. Thus, the differences in the left IFG suggest that the two groups employed different mechanisms to learn JSL.

This raises an interesting question of what the DVD Group actually learned in our experiment. On this question, the activation of the right supramarginal gyrus in the DVD Group is suggestive in terms of the result in Jeong et al. (2010): the right supramarginal gyrus is crucially involved in the retrieval of words learned by means of situation-based learning using media-clips of a dialogue. Note here that situation-based learning in Jeong et al. (2010) roughly corresponds to learning via DVD recordings in our experiment. The right supramarginal gyrus is part of the right parietal lobule, which is considered to play a key role in incorporating multimodal information from different senses (Macaluso and Driver, 2003). Jeong et al. (2010) suggest that the activation of the right supramariginal gyrus is associated with imitation learning, since the area is proposed to constitute a part of human mirror neuron systems (Chong et al., 2008). Mirror neurons are active not only during the execution of an action but also during the observation of the same action (Gallese, 2008). Learners in the DVD Group might have developed the knowledge of JSL only by observing the DVD recordings, inferring the intentions of a signer recorded there and imitating JSL to adapt to a given situation in learning sessions. The imitation of familiar gestures is also known to invoke activation in the right supramarginal gyrus (Peigneux et al., 2004). The right IFG [45, 11, 31] can also be considered the anterior component of the mirror neuron system. Putting these together, it might be reasonable to conclude that participants in the DVD Group developed the knowledge of JSL through imitation learning.

We have assumed, following the generative tradition (Chomsky, 2013a,b; Everaert et al., 2015; Berwick and Chomsky, 2016), that aside from externalization at the sensory-motor level (sign language or speech), the brain contains a universal computational system, which merges or combines smaller elements into larger elements or constituents in a hierarchical manner, generating hierarchical structures. This structure-building operation called Merge is universal, so that it does not need to be learned. If JSL and spoken Japanese differ only in “their modality of externalization” with their syntactic operations the same, one might ask what participants in the Live-Exposure Group learned. It is interesting to note here that activity in the right middle frontal gyrus ([45, 8, 37], [48, 14, 31]) in the Live-Exposure Group might show the involvement of the anterior component of the mirror neuron system, suggesting the role of the mirror neuron in the acquisition of JSL in the Live-Exposure Group. It is natural to think that the Live-Exposure Group learned JSL through observing a teacher use JSL, but second language acquisition involves much more than imitation.

Successful second language acquisition involves assembling or mapping syntactic, semantic and phonological features into new configurations, that is, second language acquisition learners are required to reconfigure features from the way they are coded in the first language into the new configuration where they are represented in the second language; this is a proposal termed “Feature Reassembly Hypothesis” (Lardiere, 2009). On this hypothesis, second language learners of JSL must develop the knowledge of which signs and non-manual markers such as facial expressions, and their variants represent which syntactic, semantic, and phonological features. In addition, they must acquire the knowledge of whether such signs are obligatory, optional or prohibited under which syntactic, semantic, phonological, lexical and pragmatic conditions (Hwang and Lardiere, 2013; Slabakova, 2016). Assuming the Feature Reassembly Hypothesis, we assume that what developed in the Live-Exposure Group is the knowledge of reassembling relevant features in spoken Japanese into new configurations in JSL by means of associating abstract features carrying grammatical information in spoken Japanese and their exponents (signs) in JSL.

To be more specific, at least two points are relevant to the question of the relation between learning second language syntactic rules and feature-reassembly. One is the knowledge of the wh-cleft in JSL and the other is the knowledge of the possessive construction in JSL.

The wh-cleft in JSL is different at least in three points from the wh-cleft in spoken Japanse (for the wh-cleft in American Sign Language, see Caponigro and Davidson, 2011). The wh-phrase in JSL must be accompanied by NMMs such as “a repeated weak headshake and furrowed eyebrows” (Matsuoka, 2015) as well as “the following fixation of the head” (Ichida, 2005). Following the analysis of wh-interrogatives in JSL by Uchibori and Matsuoka (2016), we assume that the wh-element in JSL is morphologically made up of a wh-phrase (represented by a wh-sign) and a Q-particle or a wh-interrogative marker (represented by wh-NMMs) (Uchibori and Matsuoka, 2016). The lack of these NMMs results in ungrammatical wh-cleft sentences. The wh-phrase and the Q-particle *ka* in spoken Japanese are pronounced in different positions, while in JSL the wh-phrase and the Q-particle must co-occur. Therefore, the participants had to reassembly the Q or wh-interrogative feature into the NMM in JSL and to express the wh-phrase and the NMM simultaneously. Incidentally, it is interesting to note here that *Shushi Nihongo* or *Nihongotaiou Shuwa* “Signed Japanese,” a variant of spoken Japanese, lacks NMMs (Kimura, 2011).

Second, semantics is different: the element following the wh-phrase in JSL does not receive a focus interpretation, while the counterpart in spoken Japanese is in focus. Third, pragmatics is different; the wh-cleft in JSL is commonly used and does not sound “orotund” unlike the wh-cleft in spoken Japanese (Matsuoka, 2015). These differences are what the participants learned in our experiments.

Regarding the possessive construction in JSL, nominative “I,” expressed by POINTING AT THE SPEAKER, is not accompanied by the NMM of nodding. When nodding co-occurs with pointing at the speaker, it means “and.” Thus, the difference between “my father” and “I and father” depends on the NMM (nodding). Therefore, the participants had to learn that the possessive pronoun is morphologically composed of two parts: the sign meaning the first person and the absence of nodding (NMM). It is clear that learning of the wh-cleft and the possessive construction is related to externalization, which is in turn related to the fact that a sign language can use more than one articulator simultaneously.

Second language acquisition is influenced by similarities and differences between the feature arrays charactering the first language and those in the second language input. Consequently, the magnitude of feature reassembly depends on the nature of the input: “feature reassembly may occur slowly or not at all if the relevant evidence is rare or ambiguous in the input” (Slabakova et al., 2014, p. 602). Thus, the knowledge of the association interacts with structure-building operations to result in the knowledge of specific constructions such as the possessive construction in JSL. From this perspective, it is more appropriate to say that as a result of feature reassembly the participants in Live-Exposure Group learned several constructions including the possessive construction. Even so, it is noteworthy that learning JSL through social interactions with a communicative partner had a different impact on the left IFG from learning it via DVD presentations without such interactions. Our result also suggests that the association might be influenced by the source of information, human or non-human, at least in the early stages of foreign language acquisition in adults.

We conclude the paper by pointing out four remaining issues. The first issue is concerned with the fMRI data of the Live-Exposure Group. The difference between TEST 1 and TEST 2 was found at uncorrected thresholds. One possible explanation for this result is that the Live-Exposure Group had already learned the possessive construction at the time of TEST 1, which was conducted just after the fourth class; knowledge of the possessive construction at TEST 1 could have washed away clear instruction effects at TEST 2, leading to the result at uncorrected thresholds. Had TEST 1 been carried out before the instruction of JSL started, more significant results at corrected thresholds should have been obtained.

The second concerns the behavioral results in TEST 2 obtained just after the tenth class, which did not show any significant differences in error rates between the Live-Exposure Group and the DVD-Exposure Group. This result seems strange but it is consistent with previous research showing that the same performance outcomes do not show the use of the same brain system (Poldrack et al., 2001; Foerde et al., 2006; Morgan-Short et al., 2012). Greater changes in the brain may be needed to show the corresponding changes in the behavior (Boyke et al., 2008). It is not clear from our experiment whether knowledge acquired from DVD-Exposure learning is as durable as knowledge obtained from social interactions with a deaf signer. The impact of social interaction on the long-term retention of newly acquired knowledge in adults is an issue for future research.

The third has to do with interactive learning tools such as video chatting with the properties of social interactions and video, as well as interactive media such as Skype or FaceTime. The interactive situation resembles a natural learning situation between a teacher and a student. Positive effects of interactive media use on second-language learning, if confirmed, will provide new insights into the issue of quality and quantity of input in second-language learning, thereby rethinking the issue of critical or sensitive periods in second-language learning. In birds, richer social interaction can delay the critical period closure for learning (Brainard and Knudsen, 1998). Even adults beyond sensitive periods in second language acquisition may also benefit from richer social interaction (Zhang et al., 2009). In addition to the quantity of input, its quality, not age, matters in the attainment of native-like processing of a second language (Piske and Young-Scholten, 2009).

The last is concerned with the relation of linguistic experience (input) and innate mechanisms in language acquisition. Whatever approaches to language acquisition, there is some consensus that language grows in the brain from the interaction of several factors, including at least three factors: genetic endowment (innate mechanisms), experience (linguistic input) and language-independent properties (Chomsky, 2005; Everaert et al., 2015). Although the importance of the second factor (input) for the ontogenesis of language in an individual is not controversial, what properties are attributed to innate mechanisms characterize two approaches to language acquisition. One approach (called generative approach) assumes that a human is born with the language-dedicated cognitive system (called Universal Grammar), which grows into knowledge of a particular language through the interaction of linguistic experiences; the other (called a general or nativist emergent approach) denies a language-specific innate mechanism, but instead proposes the innate domain-general learning mechanism including statistical learning. On the latter account, linguistic knowledge emerges as a result of linguistic experiences or linguistic usage through statistical learning (O'Grady, 2005). However, the current minimalist program in generative grammar has dramatically minimized the innate language-specific properties by reducing them to other cognitive systems (see Chomsky, 2005, 2013a,b). As a result, the two approaches just mentioned are not as mutually exclusive as they used to be (Yang, 2004; Kirby, 2014). Further research needs to examine whether and to what extent the two approaches converge. It should be noted that generative grammar has never claimed that social interaction or frequency of words is not responsible for language acquisition. Then, what effects does social interaction have on second language acquisition? Our data show that learning second language syntax in social and non-social contexts can lead to differences in brain processing that cannot be reflected by behavioral data. Future research will be needed to characterize the details of the relationship between social interaction and adult second language learning, and thereby to maximize the brain development responsible for learning.

## Conclusion

The current study investigated effects of social interaction on the acquisition of syntax in adult second language learners. We found that learning JSL through interactions with a deaf signer resulted in a stronger activation of the left IFG than learning through identical input via DVD presentations, though behavioral data did not show distinct differences. This study provides the first neuroimaging data to show that interaction with a human being aids acquiring syntactic rules and in turn causes significant changes in the brain. If the activation in the left IFG is indicative of native-like processing of syntax, one implication for second language learning is that learning second language syntax in a richer social context may well lead to native-like attainment of second language processing. This implication calls for further studies on whether interactive media such as Skype or FaceTime will induce distinct changes than traditional learning media such as DVDs and TV programs.

## Author contributions

NY contributed to the experimental design. NY, JK, and MK created experimental materials and conducted the experiment with MS and RK. JK analyzed the data. NY wrote the manuscript with advice from JK, MK, and MK, except for the Image Acquisition and Analysis sections, which JK wrote. JK prepared the figures. NY financially supported the experiment.

## Funding

This work was supported in part by the Grants-in-Aid for Scientific Research on Priority Areas (#20020022, Noriaki Yusa, PI), Challenging Exploratory Research (#25580133, 16K13266, Noriaki Yusa, PI) from the Japan Society for the Promotion of Science, and the 2010 Special Research Grant from Miyagi Gakuin Women's University (Noriaki Yusa, PI).

### Conflict of interest statement

The authors declare that the research was conducted in the absence of any commercial or financial relationships that could be construed as a potential conflict of interest. The reviewer VH and handling Editor declared their shared affiliation, and the handling Editor states that the process nevertheless met the standards of a fair and objective review.
